# Clinical Advancements in the Targeted Therapies against Liver Fibrosis

**DOI:** 10.1155/2016/7629724

**Published:** 2016-11-24

**Authors:** Ruchi Bansal, Beata Nagórniewicz, Jai Prakash

**Affiliations:** Targeted Therapeutics, Department of Biomaterials Science and Technology, Faculty of Science and Technology, University of Twente, Enschede, Netherlands

## Abstract

Hepatic fibrosis, characterized by excessive accumulation of extracellular matrix (ECM) proteins leading to liver dysfunction, is a growing cause of mortality worldwide. Hepatocellular damage owing to liver injury leads to the release of profibrotic factors from infiltrating inflammatory cells that results in the activation of hepatic stellate cells (HSCs). Upon activation, HSCs undergo characteristic morphological and functional changes and are transformed into proliferative and contractile ECM-producing myofibroblasts. Over recent years, a number of therapeutic strategies have been developed to inhibit hepatocyte apoptosis, inflammatory responses, and HSCs proliferation and activation. Preclinical studies have yielded numerous targets for the development of antifibrotic therapies, some of which have entered clinical trials and showed improved therapeutic efficacy and desirable safety profiles. Furthermore, advancements have been made in the development of noninvasive markers and techniques for the accurate disease assessment and therapy responses. Here, we focus on the clinical developments attained in the field of targeted antifibrotics for the treatment of liver fibrosis, for example, small molecule drugs, antibodies, and targeted drug conjugate. We further briefly highlight different noninvasive diagnostic technologies and will provide an overview about different therapeutic targets, clinical trials, endpoints, and translational efforts that have been made to halt or reverse the progression of liver fibrosis.

## 1. Liver Fibrosis: Mechanism and Pathogenesis

**Figure 1:**
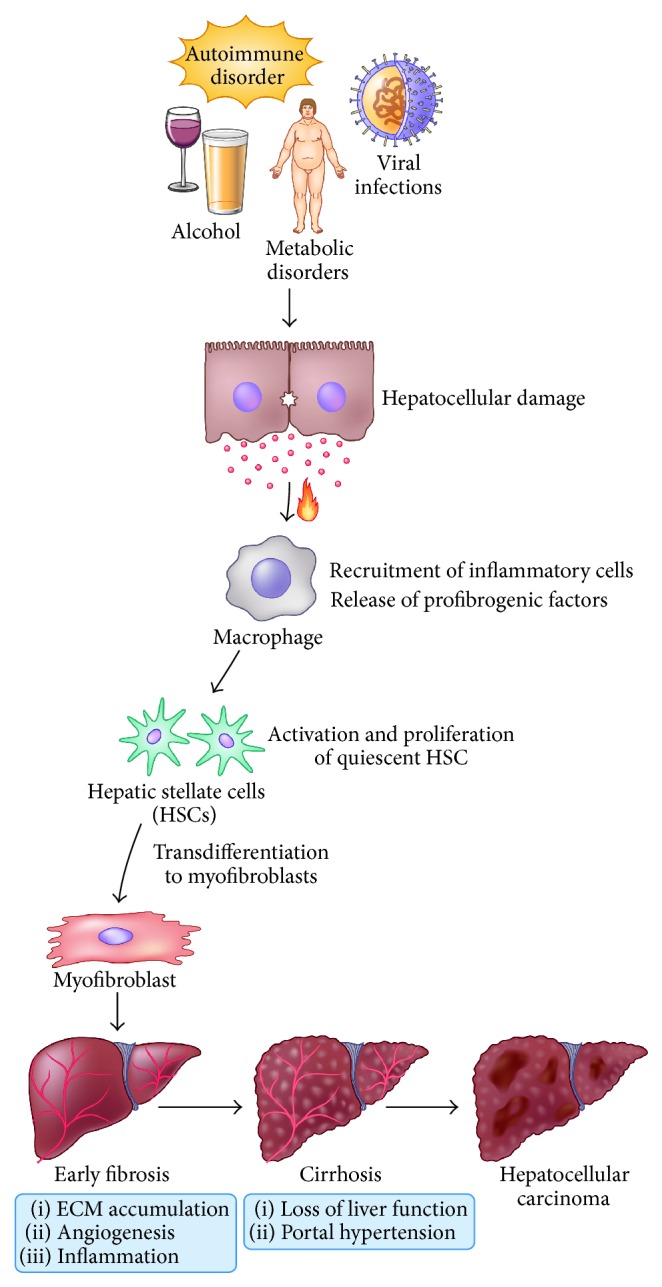
Hepatic injury initiated by chronic viral infections, excessive alcohol consumption, metabolic disorders, or autoimmune insult leads to the development of liver fibrosis. Hepatocellular damage instigates the recruitment of inflammatory cells and release of profibrogenic factors that result in the transdifferentiation of the resident quiescent liver fibroblast (hepatic stellate cells, HSCs) to the highly activated, proliferative, motile, and contractile myofibroblast phenotype. ECM accumulation, angiogenesis, and inflammation lead to progressive fibrosis ultimately culminating into cirrhosis associated with loss of liver function and portal hypertension, or hepatocellular carcinoma.

Hepatic injury of various etiologies, such as chronic viral infections (mainly HCV and HBV), excessive alcohol consumption, metabolic disorders, or autoimmune insults, leads to the development of liver fibrosis. Fibrosis is a prolonged and exorbitant wound healing response causing the accumulation of redundant extracellular matrix (ECM). ECM consists of a dense mesh of macromolecules, polysaccharides, and proteins, particularly *α*-smooth muscle actin and different types of collagen, forming insoluble fibers and microfibrils. Its main function is to support the structure and functioning of the tissue during healing processes. In the physiological state, balance between ECM deposition and degradation is controlled by numerous matrix metalloproteinases (MMPs), which are digesting/degrading the particular components of ECM. In the course of fibrosis, however, MMPs are markedly inhibited by tissue inhibitors of MMPs (TIMPs) that are upregulated in response to the chronic liver insult. The organ is progressively hardening and stiffening and its physiological functions are hindered. Continuous scarring may eventually lead to the development of liver cirrhosis, end-stage liver disease, or hepatocellular carcinoma [1, 2] (Figure 1).

*Myofibroblasts: Primary Source of Fibrosis.* Hepatic injury initiates cascade of fibrogenic processes initiated by inflammatory and fibrogenic signals. These fibrogenic stimuli include reactive oxygen species (ROS), hypoxia, inflammatory and immune responses, hepatocytes apoptosis, and steatosis. Response to these signals owing to persistent liver injury instigates the recruitment and transformation of the resident quiescent liver fibroblast (hepatic stellate cells, HSCs) to the highly activated, proliferative, motile, and contractile myofibroblast phenotype (Figure 1). Myofibroblasts are the main source of the excessive ECM responsible for the liver fibrosis. The activation process is initiated by the release of many growth factors such as platelet-derived growth factor (PDGF) and transforming growth factor *β* (TGF-*β*), profibrogenic cytokines and chemokines by the injured hepatocytes, and inflammatory cells particularly macrophages and other nonparenchymal cells. Deposition of the dense and complex net of scar tissue in the space of Disse, where HSCs reside, causes significant changes in the sinusoid architecture. Fenestrations in the structure of liver sinusoidal endothelial cells (LSECs) are gone and hepatocytes lose their microvilli. Moreover, contractile activated HSCs contribute to portal hypertension. Although HSCs remain the primary source of myofibroblasts, it has now become clear that other cell types can also contribute to myofibroblasts population including portal fibroblasts, bone-marrow derived cells, and possibly epithelial-mesenchymal transition (EMT) and contribute to the liver scarring. However, recruitment of these different myofibroblastic cells might be potentially disease-specific. Liver fibrosis is clinically silent, slowly progressive, and mostly asymptomatic disease. First symptoms of the liver impairment in most of cases are indicating disease development into cirrhosis and this commonly occurs after 15–20 years, when the prognoses of survival and recovery are dramatically reduced. The only effective treatment for end-stage liver failure is liver transplantation.

## 2. Assessment of Liver Fibrosis

A major difficulty in developing disease-specific therapy is the lack of accurate and established diagnostic techniques for long-term monitoring of disease progression and therapy responses and to optimize disease treatment strategies [3, 4]. Liver biopsy has been considered as the* gold standard* for the diagnosis and staging of liver fibrosis but is invasive and painful and has numerous limitations including risk of bleeding, sampling errors due to disease heterogeneity, and inter- and intraobserver variability [4–6]. Moreover, liver biopsies only sample 1/50,000 of the liver, and undersized or fragmented samples may therefore underestimate hepatic fibrosis [4, 5, 7]. Recently, guidelines by EASL-ALEH have been published summarizing and validating clinical use of noninvasive tests for evaluation of liver disease severity and prognosis [8].

### 2.1. Class I and Class II Biomarkers

The tremendous advancement in the biomedical research over the last decade led to the development of novel, rapid blood tests for diagnosis of liver fibrosis. Several commercial biochemical and serum tests classified into Class I and Class II biomarkers are developed. Class I biomarkers are associated with the mechanism of fibrogenesis, either as secreted matrix-related components or as a result of ECM synthesis or turnover, for example, Hyaluronan. Class II biomarkers are indirect methods which are grouped into panels such as (a)* European liver fibrosis test (ELF)* (N-terminal propeptide of collagen type III, hyaluronic acid, TIMP1, and age), (b)* Fibrotest* (Alpha-2-macroglobulin, Haptoglobin, Apolipoprotein A1, Gamma-glutamyl transpeptidase [GGT], total bilirubin, and Alanine transaminase), (c)* fibrosis-4 index (FIB-4)* combining standard biochemical tests (platelets, ALT, and AST) and age, (d)* HepaScore* (age, sex, total bilirubin, Gamma-glutamyl transferase, 2-macroglobulin, and hyaluronic acid), (e)* aspartate and transaminase to platelet ratio (APRI)*, and (f)* Forns score* (platelet count, prothrombin index, AST, Alpha-2-macroglobulin, HA, and blood urea) which have been developed recently [9–13]. However, these tests rely on indirect markers and lack specificity as these markers can be influenced by unrelated diseases [14]. Nevertheless, recent studies indicate that the results from the serum panels might predict risk of decompensation and overall survival more accurately than biopsy [9, 10, 12].

### 2.2. Noninvasive Imaging Modalities

Number of emerging technologies have recently been developed for diagnosing and staging liver fibrosis over the past years such as ultrasonography (US), computerized tomography (CT), and magnetic resonance imaging (MRI). However, these imaging modalities are dependent primarily on structural and morphological alterations in the liver and these alterations are usually identified in advanced stage of fibrosis [14]. Currently,* transient elastography (TE)* (Fibroscan, EchoSens, Paris, France) is the most widely used method for noninvasive and rapid measurement of liver stiffness. TE uses a probe consisting of an ultrasonic transducer and a vibrator that emits low-frequency shear waves (50 Hz) propagating through the liver tissue. The speed of the shear waves is directly related to liver stiffness and can be expressed in kiloPascal (kPa). Several studies have evaluated TE for diagnosis of hepatic fibrosis and cirrhosis with relatively high specificity and sensitivity [15–18].* Point Shear wave elastography* (pSWE) or* acoustic radiation force impulse (ARFI)* involves mechanical excitation of tissue using short-duration acoustic pulses that produce shear waves, expressed in m/sec, which directly correlates with the extent of liver fibrosis [19–25]. Another promising technique,* 2-dimensional shear wave elastography (2D-SWE),* is based on the combination of a radiation force induced in tissues by focused ultrasonic beams and a very high frame rate ultrasound imaging sequence capable of catching in real time the transient propagation of resulting shear waves [26]. 2D-SWE expressed either in m/sec or in kPa has an advantage of being implemented on a commercially available ultrasound machine.

New magnetic resonance imaging (MRI) based imaging techniques have recently gained substantial interest:* magnetic resonance elastography (MRE)*,* dynamic contrast-enhanced MR imaging (DCE-MRI)*,* perfusion weighted imaging (PWI),* and* diffusion weighted imaging (DWI)* [27–29]. Magnetic resonance elastography (MRE) is similar to ultrasound based elastography techniques and can determine liver stiffness by analysis of mechanical waves propagating through the liver [30–34]. Diffusion weighted imaging (DWI) is a magnetic resonance technique that quantifies the diffusion of water molecules in tissues that can be quantified as apparent diffusion coefficient (ADC). Collagen fibers in the liver would inhibit water diffusion thereby leading to a decrease in ADC and therefore can be quantitatively used to assess liver fibrosis, but the technique has limitations since factors like steatosis can also affect ADC [35]. Dynamic contrast-enhanced MR imaging (DCE-MRI) and MR perfusion weighted imaging (MR-PWI) rely on the intravenous administration of MR contrast agents that can more precisely reveal hepatic hemodynamic changes [36–38]. However, these MRI-based techniques are time-consuming and cost-ineffective. Another novel and developing MR based imaging modality,* molecular MR imaging*, represents a unique implementation of MR modality to visualize, characterize, and measure biological processes at the cellular and molecular level with high spatial resolution. The specific contrast agents (or probes) can be endogenous and exogenous probes can be generated by encapsulating paramagnetic (Gadolinium) or superparamagnetic (iron-oxide) metals in different nanoparticles. Molecular MR imaging is based on the development of MR imaging probes composed of contrast generating materials, for example, Gadolinium or iron-oxide, and molecular targets, for example, ECM binding probes such as collagen I (EP-3533), fibrin-fibronectin (CLT1-peptide), Elastin (EMSA), and *α*v*β*3-Integrin (c(RGDyC)-USPIO) [39, 40].

Overall, no single method can provide the detailed information as histological examination but using noninvasive modalities can differentiate between mild and significant fibrosis and can potentially avoid unnecessary liver biopsy in a subgroup of patients. While these methods have provided some impressive results, there remains a paucity to validate their use in disease management or assessment of potential antifibrotic therapies. Although molecular MRI of liver fibrosis is currently developing, the conception of target specific molecular MRI approach can open up new horizons and avenues for the diagnosis and effective management of this life-threatening disease.

## 3. Approaches for Targeted Therapy

The term “targeted therapy” (TT) describes the set of treatment strategies aiming to inhibit or alter specific molecules or molecular pathways leading to certain disorders and diseases. Some of the molecularly targeted agents exert the cytotoxic or cytostatic effects on the specific target cell types, while others inhibit the activity of the particular enzymes or proteins or boost the immune system activity against pathogenic mechanism. One of the main advantages of such approach is its specificity—the principle of design is to affect only the pathologically transformed cells and processes, thus minimizing the adverse effects [41, 42].

Most important part of the targeted therapy development is to determine the appropriate molecular target proteins and enzymes, hormones, peptides, genes, and specific reactions involved in the pathological processes that, upon alteration, can lead to the disease resolution/reversion [42]. Three main types of the targeted therapy design can be distinguished:

- *Small molecule drugs*: relatively small moieties which are able to target molecules and processes inside the cell [43–46]
- *Monoclonal antibodies*: large proteins produced by the immune cells that are able to highly specifically identify and bind with the targets on the cell surface or outside the cells [47–49],
- *Targeted conjugates*: delivery systems consisting of the therapeutic moiety, such as delivery vehicle or protein carrying therapeutic agent conjugated with the targeting ligands [50–52]

The antifibrotic therapeutic approaches are broadly classified among several categories:

- Elimination of the primary cause of injury, for example, alcohol abstinence in alcoholic liver diseases
- Reduction of inflammation and immune response or inhibition of hepatocyte apoptosis/injury to avoid HSC activation
- Resolution of fibrosis by inhibiting scar tissue formation, increasing matrix degradation, inhibiting HSC activation, or stimulating HSC apoptosis
- Inhibition of signaling pathways (extracellular and intracellular) responsible for activation, contraction, and proliferation of HSCs

## 4. Current Clinical Studies Overview

There is an intensified focus on the development of antifibrotic therapies for chronic liver diseases in the past years. A remarkable number of clinical trials worldwide have been carried out. Advanced pathological and molecular understanding of the fibrosis pathogenesis has instigated identification of novel therapeutic and promising drugs in preclinical models. Furthermore public health impact of liver diseases and novel diagnostic technologies for the assessment of fibrosis has resulted in increased clinical trials in this field [53]. In this review, clinical studies concerning targeted therapies against liver fibrosis of diverse etiology are reviewed and summarized in Table 1. In general, the biggest emphasis is on the small molecule drugs; so far these therapeutics were the most frequently investigated and the progress in this field is currently the most advanced. Reviewed studies mostly are randomized trials on the parallel two or more groups of patients (parallel assignment design); less frequently there are also single group assignments. Most of the studies are randomized and double-blinded to ensure the minimal risk of the results manipulation or bias. Clinical trials are mostly performed on patients with NASH (nonalcoholic steatohepatitis), liver fibrosis, or cirrhosis with chronic hepatitis C infection and NAFLD (nonalcoholic fatty liver diseases) since these diseases are the most frequently occurring reasons for the development of liver fibrosis. Clinical trials in chronic liver diseases present unique challenges, because clinical events that could be used as trial primary endpoints (e.g., histological assessment of fibrosis) can vary depending on the etiology of the liver disease; therefore the study outcomes largely rely upon noninvasive surrogates. Current clinical trials are primarily based on pathological characterization of liver biopsy to assess fibrosis progression but now serum tests such as HepaScore, ELF, Fibrotest and noninvasive imaging modalities like TE or MR are characterized as surrogate endpoints [53]. In the following list, we have summarized the clinical endpoints used in the clinical trials.

*Liver Histology*

- Necroinflammation: NAFLD activity score and Knodell score
- Fibrosis: histopathological and immunohistochemical analysis

*Serum Tests*

- Serum markers: ALT, AST, ALP, GGT, and albumin
- Serum marker panels: ELF test, APRI, and FIB-4
- Lipidomic analysis

*Liver Function Tests*

- Insulin sensitivity
- Glucose tolerance
- Indocyanine green clearance tests
- Galactose elimination tests

*Noninvasive Tests*

- Liver stiffness measurement: transient elastography (Fibroscan); shear wave elastography; magnetic resonance elastography; acoustic radiation force impulse (AFRI)
- Liver fat measurement: MRI and spectroscopy (MRS)

*Clinical Scores*

- MELD score
- Child-Pugh score
- Ishak score
- Metavir score.

## 5. Developments in Targeted Therapy Related to Liver Fibrosis

### 5.1. Small Molecule Drugs

Small molecule drugs are the group of the targeted therapeutic agents typically with molecular weight below 1000 Da. They can be delivered intravenously or orally and, due to their small size, enter the target cells (cross the cell membrane); typically they are also able to penetrate the blood-brain barrier. The complex process of discovery and development of small molecule drugs mostly consists of two combined strategies: (I) knowledge-based design employing the knowledge about the structure of the target and its inhibitors/ligands and/or (II) random high throughput screening of libraries of small molecules to search for the molecules with potential activity towards/against the target. Following extensive screening, the identified promising molecules are evaluated for selectivity and potency. Eventually, the prospective compounds are further investigated* in vitro* and* in vivo* for the therapeutic efficacy and, if applicable, enter further the clinical development phase [54, 55]. Some of the major clinically challenged targets of the small molecule drugs are mentioned below.

#### 5.1.1. Nuclear Receptors

Activated HSCs express a diverse group of nuclear receptors acting as transcription factors, for example, peroxisome proliferator-activated receptor *γ* (PPAR*γ*) and Farnesoid X receptor (FXR), that play an important role in HSC regulation [56]. PPAR*γ* is highly expressed in the quiescent HSCs and upon activation its expression diminishes [57]. Following treatment with PPAR*γ* ligands/agonists, PPAR*γ* expression is restored, and HSC activation and collagen expression are reduced* in vitro* [58]. Clinical trials using pioglitazone showed significant improvement in steatosis, inflammation, and insulin resistance in NASH patients [59, 60] (Table 1), while clinical trials using PPAR*γ* agonists Farglitazar (GI262570) [61, 62] showed no effective treatment in patients with chronic HCV infection (Table 1).

FXR, another nuclear receptor, is highly expressed in the liver and small intestine. It is responsible for maintaining homeostasis of bile acids and cholesterol and regulates transcription of multiple genes involved in bile acids synthesis and transport [63]. FXR is also expressed in HSCs and activation of FXR in HSCs is associated with significant decrease in collagen production [64]. Activation of FXR occurs via binding with bile acids such as deoxycholic or lithocholic acid, although many synthetic ligands are also known [65]. However, most FXR ligands failed the preclinical and clinical assessment because of poor pharmacokinetics or toxicity issues. Nevertheless, synthetic FXR agonists Px-102 and Px-104, developed by Phenex Pharmaceuticals, showed promising safety and tolerability profile in healthy subjects (Px-102, clinical trial NCT01998659; NCT01998672 [66]) and Px104 is currently tested in a phase 2a study in patients with NAFLD (NCT01999101). INT-747 (6*α*-Ethyl Chenodeoxycholic Acid or 6-ECDCA or obeticholic acid), semisynthetic FXR agonist, showed improvement of the histological and biochemical markers, ameliorated fibrosis, inflammation, and steatosis in NASH patients [67]. Obeticholic acid is currently in clinical trials for long-term treatment of cholestatic liver diseases (Table 1).

#### 5.1.2. Renin-Angiotensin System (RAS)

RAS is an important hormonal regulatory mechanism of the blood pressure and body fluid homeostasis. Several studies have shown upregulation of RAS activity during liver fibrosis [68]. The key RAS protein, angiotensin II (Ang II), is produced in the liver from its precursor angiotensin I by the proteolytic cleavage by angiotensin I converting enzyme (ACE) [68]. Ang II exerts its diverse biological effects by binding with one of its multiple receptors, particularly Ang II type 1 receptor (AT1-R), overexpressed in activated HSCs [69]. Ang II induces HSC activation, proliferation, and contraction [70], as well as increased TGF*β*, TIMP1 expression, and collagen deposition [68]. Finally, Ang II also contributes to the oxidative stress in the fibrotic liver. Therefore, Ang II and its interaction with AT1-R are considered to play an important role in liver fibrogenesis and its blocking by ACE inhibitors (ACEi) or AT1-R blockers (ARBs) may be an effective therapeutic option for treatment of liver fibrosis and they are already in clinical trials, for example, Losartan [71], Irbesartan [72], and Candesartan and Moexipril [73] (Table 1). Losartan, Irbesartan, and Candesartan share similarities in the chemical structure and they all are AT1-R blockers, in contrast to Moexipril, which is an ACE inhibitor.

Clinical trials evaluating long-term Losartan effects in chronic hepatitis C patients showed decreased inflammation, reduced expression of fibrogenic mediators, and decreased ECM (collagen I) accumulation [71, 74]. Furthermore, treatment markedly decreased Ang II induced oxidative stress in hepatic fibrosis [71, 74]. Prolonged exposure to the AT1-R blocking treatment in patients with chronic HCV infection was proven to be safe and well tolerated. RAS has been also shown to be associated with hypertension; therefore, Candesartan (AT1-R inhibitor), widely used for the therapy of hypertension and heart failure, has shown promising results in the clinical trials for alcoholic liver fibrosis in combination with ursodeoxycholic acid (UDCA). It was demonstrated that Candesartan significantly improved the treatment outcomes in comparison to UDCA and reduced the fibrosis scores and *α*-SMA positive fibrotic area in biopsies. Relative expression of fibrogenic markers was downregulated and the arterial blood pressure was shown to be significantly reduced [75]. However, long-term treatment with Irbesartan (ARB and antihypertensive drug) in severe fibrosis with chronic hepatitis C showed no substantial improvement in fibrosis scores, arterial pressure, and organ stiffness in the treated group, despite the fact that treatment was safe and well tolerated [72]. In addition, ACE inhibitor Moexipril treatment did not show beneficial effects in primary biliary cirrhosis patients [73]. Furthermore, in HALT-C cohort study, ACEi/ARB therapy did not retard the progression of fibrosis [76]. Due to ambiguous results, further controlled studies are required to evaluate the long-term efficacy of ARBs/ACEi.

#### 5.1.3. Endocannabinoid System

Endocannabinoid system plays an important role in various liver diseases including viral hepatitis, NAFLD, and alcoholic liver disease. Cannabinoid receptors CB1 and CB2 are upregulated in chronic liver diseases and several studies have convincingly demonstrated antagonism between CB1 and CB2; that is, CB1 promotes while CB2 suppresses liver damage [77, 78]; therefore CB1 antagonists and CB2 agonists were investigated as potential therapeutic approaches for liver diseases. Clinically, daily cannabis (CB1 and CB2 agonist) promoted fibrosis progression in chronic hepatitis C [79]. Rimonabant CB1 antagonist was successfully tested in clinical trials in obese patients and showed reduction in body weight, improved metabolic function, and improved insulin resistance [80]. However, depression and psychoactive side effects led to the termination of clinical Rimonabant drug use. Currently, efforts are directed towards development of novel CB1 antagonist with improved specificity that lacks neuropsychiatric adverse effects. Other neurotransmitters, for example, opioids and serotonin (5HT), and their receptors are other potential therapeutic targets in liver fibrosis. Opioid antagonist Naltrexone and 5HT antagonist Methiothepin have shown antifibrotic activity in animal models of liver disease [81, 82], but clinical trials are needed to demonstrate their long-term tolerability and efficacy.

#### 5.1.4. Inflammation and Oxidative Stress

Since inflammation promotes progression of liver fibrosis, use of anti-inflammatory drugs poses a potential and rationale therapeutic approach. Corticosteroids (e.g., prednisone, prednisolone, methyl prednisone, and triamcinolone) are used for the treatment of liver diseases, most commonly autoimmune hepatitis with improved outcome and survival. Corticosteroids are also used after liver transplantation to prevent rejection. However, the adverse effects of long-term corticosteroid therapy are still the major causes of morbidity and mortality [83]. Another anti-inflammatory approach is to inhibit release of inflammatory cytokines or to neutralize it with receptor antagonists. Upregulated TNF*α* production is one of the initiating events in the liver injury leading to release of proinflammatory cytokines resulting in fibrosis. Pentoxifylline (PTX) is a potent phosphodiesterase inhibitor, which suppresses tumor necrosis factor *α* (TNF*α*) production. PTX was also shown to be hepatoprotective since it reduces oxidative stress, which is important contributor in the hepatic pathologies and fibrogenesis [84]. PTX has been registered for numerous clinical trials concerning its potential therapeutic efficacy in diverse fibrotic disorders [75, 85–88]. Long-term treatment with PTX in NASH patients demonstrated significant improvement of both histological features and significant improvement in the liver fibrosis in comparison to placebo-treated group [89]. Despite the fact that PTX activity is being associated with TNF*α* inhibition, the study failed to demonstrate the TNF*α* downregulation. Finally, it was also concluded from the study that PTX treatment was safe and well tolerated by patients and there were no severe adverse side effects [89].

Following hepatocyte injury, hepatic macrophages secrete inflammatory chemokines or cytokines, for example, C-C chemokine ligand type 2 [CCL2 or MCP1 (monocyte chemoattractant protein-1)], driving the recruitment and migration of pro-CCR2 and CCR5 positive inflammatory monocytes to the liver [90]. CCR2 and/or CCR5 antagonism has been suggested as a potential approach for the treatment of inflammatory diseases and fibrosis [91, 92]. Cenicriviroc (CVC), a CCR2/CCR5 antagonist, is currently being evaluated for the treatment of NASH and liver fibrosis (CENTAUR, NCT02217475, Table 1). Cenicriviroc showed favorable safety profile in HIV-infected patients in a phase 2b study [93] and in patients with hepatic impairment [94].

Another target molecule is Galectin-3 (Gal-3), pleiotropic *β*-galactoside-binding lectin, that was shown to play an important role in the liver fibrosis. Gal-3 possesses strong proinflammatory properties and is able to activate macrophages and stimulate their migration. Furthermore, Gal-3 stimulates HSC proliferation via ERK1/2 dependent pathway. Gal-3 knockout mice exhibited constricted susceptibility to the CCl_4_-induced liver fibrosis [95]. GR-MD-02 (galactoarabino-rhamnogalacturonate) is a potent inhibitor of Galectin-3 [96] that showed remarkable therapeutic effects in thioacetamide-induced liver fibrosis in rats [97] and was submitted for 3 clinical studies concerning liver fibrosis. Phase 1 study evaluating safety of GR-MD-02 in patients with nonalcoholic steatohepatitis (NASH) and advanced fibrosis is already completed [65, 98, 99]. Results showed that the drug was safe and well tolerated in NASH patients with liver fibrosis and demonstrated improvement in fibrosis and inflammation [100–102]. Two upcoming clinical trials will evaluate GR-MD-02 efficacy for the treatment of liver fibrosis in patients with advanced fibrosis [65] and cirrhosis [99] originating in NASH.

Oxidative stress or reactive oxygen species (ROS) generation also plays an important role in initiation of fibrogenesis by activation of HSCs; therefore inhibition of oxidative stress or ROS inhibits inflammation resulting in amelioration of liver fibrogenesis. Antioxidants can attenuate ROS generation and therefore emerge as potential antifibrotic therapies. Hence, a number of antioxidants, for example, S-adenosyl-L-methionine (SAMe), silymarin, phosphatidylcholine, N-acetylcysteine (NAC), and vitamin E, are and have been tested in clinical trials (refer to Table 1) with beneficial effects [59].

#### 5.1.5. Protein Kinases/Kinase Receptors

During liver fibrosis, a number of receptor tyrosine kinases, that is, PDGFR (platelet-derived growth factor receptor), VEGFR (vascular endothelial growth factor receptor), FGFR (fibroblast growth factor receptor), and EGFR (epidermal growth factor receptor), were significantly upregulated on activated HSCs. Many fibrotic and proliferative cytokines, for example, PDGF, TGF, FGF, and VEGF, signal via these receptors tyrosine kinases resulting in the activation of intracellular signaling pathways resulting in differentiation and proliferation of quiescent HSCs [2, 103–105]. Antagonism of these pathways via tyrosine kinase inhibitors attenuates liver fibrosis in preclinical experiments on animal models [106].

Sorafenib, multitargeted tyrosine kinase inhibitor, was shown to attenuate liver cirrhosis, portal pressure, and angiogenesis [107]. In a pilot clinical trial, sorafenib showed beneficial effect on portal hypertension in patients with cirrhosis [108]. Recently, multicentered randomized clinical trial was carried out to study the effect of sorafenib on portal pressure in patients with cirrhosis (NCT01714609, Table 1). Erlotinib, EGFR kinase inhibitor, attenuated liver fibrosis and HCC development in experimental animal models by suppression of EGFR phosphorylation and inhibition of HSC activation [109]. Currently, clinical trial is ongoing to evaluate the effects of erlotinib in inhibition of fibrogenesis and HCC prevention (NCT02273362, Table 1).

Apoptosis signal-regulating kinase 1, ASK1, a serine/threonine kinase, promotes oxidative stress responsive pathway and leads to the activation of downstream p38 mitogen-activated protein kinases (MAPK) and c-Jun N-terminal kinase (JNK), which stimulates inflammatory cytokines production, matrix remodeling genes expression, and abnormal cell proliferation. ASK1 and p38 have been positively correlated with the fibrosis stage in patients with NAFLD. Selonsertib (or GS-4997) is a highly selective and potent (ASK1) inhibitor [110] that inhibits ASK1 by competitive binding to the catalytic domain of ASK1 [111].* In vivo* in murine model, treatment with GS-4997 reduced fibrosis and steatosis, thus ameliorating the liver disease [112], thereby suggesting ASK1 inhibition as the promising therapeutic approach. Pharmacokinetics of GS-4997 have been already evaluated in phase 1 clinical study in adult with normal or impaired liver function (NCT02509624) and are currently registered in other clinical trials (Table 1).

Mammalian target of rapamycin (mTOR) is a serine/threonine protein kinase that is able to regulate cell growth and proliferation by controlling the protein translation [113]. mTOR performs its action by formation of mTOR complexes 1 and 2 (mTORC1 and 2) that further transmit the signal to the downstream effector proteins, that is, ribosome kinase p70S6 and 4E-BP1, which are directly responsible for mRNA translation. During fibrosis, mTOR is highly dysregulated and was shown to be involved in the TGF*β* responsiveness of the fibroblasts [114]. Therefore, mTOR inhibition represents a promising approach in liver fibrosis amelioration. mTOR inhibitors impair the mTOR by compromising the mTORC1 formation [115]. mTOR inhibitors were first shown to possess immunosuppressive properties and to date they are used as the immunosuppressive drugs preventing posttransplant organ rejection as well in autoimmune diseases (e.g., rheumatoid arthritis). They can also benefit treatment of several neoplastic malignancies [115]. The foremost mTOR inhibitor is rapamycin (or sirolimus); however due to its stability and solubility issues, new derivatives have been developed with improved safety and pharmacokinetics. Everolimus, one of the above-mentioned analogues, has been investigated in patients after liver transplantation [116, 117].

### 5.2. Monoclonal Antibodies

Monoclonal antibodies (mAbs) are very relatively recent and becoming an essential element of the present pharmacotherapy [41, 47]. Utilization of mAbs causes less adverse side effects and, alone or in combination with other drugs, can give remarkable results. There are many clinically approved mAbs therapies for different types of diseases used either as monotherapies or as combined treatments (e.g., cetuximab [118], herceptin with docetaxel or paclitaxel [41]). Monoclonal antibodies are rather new approach in the liver fibrosis treatment; therefore the development of the field is relatively in early stage. Nevertheless, several formulations reached clinical assessment.

Connective tissue growth factor (CTGF) is a heparin-binding ECM-associated protein, highly upregulated during liver injury. CTGF is synthesized by fibroblasts and promotes the proliferation and migration of these cells. It stimulates ECM deposition (particularly collagen I and fibronectin) and is involved in ECM remodeling [103], important features of liver fibrosis. Monoclonal antibody against CTGF (FG-3019) was developed by FibroGen for treatment of the fibrotic disorders. FG-3019 was investigated in idiopathic pulmonary fibrosis (IPF) patients, and, after 2 years of the treatment, FG-3019 was proven safe and well tolerated in IPF patients [49]. FG-30149 is recently being tested in phase 2 trials in subjects with liver fibrosis as a result of a chronic hepatitis B infection [119].

Vascular adhesion protein-1 (VAP1) is an endothelial glycoprotein that promotes leukocytes trafficking from the blood to the site of inflammation. Upon injury and inflammation, VAP1 translocates from intracellular storage to the cell surface. Soluble form of VAP1 (sVAP1) is also able to initiate oxidative stress and secrete, via NF*κ*B, potent proinflammatory mediators. It was shown that serum levels of sVAP1 are markedly elevated in patients with chronic inflammatory liver diseases [120]. Blockade of VAP1 inhibits inflammatory responses by attenuating leukocyte recruitment and oxidative stress [120, 121]. BTT-1023, a human monoclonal antibody against VAP1, will be assessed in the clinical study in patients with primary sclerosing cholangitis. This autoimmune liver disease is characterized by the progressive destruction of the hepatic bile ducts, which in turn leads to liver fibrosis and cholestasis [122]. Preclinical studies showed efficient binding of the antibody with VAP1 in the inflamed sites* in vivo*, as assessed by PET scans [121].

Elevated levels of lysyl oxidase-like-2 (LOXL2) expression were found in patient samples from liver fibrosis and primary biliary cirrhosis; moreover it was found that the upregulation of LOXL2 is limited to the fibrotic areas [145]. LOXL2 is an copper-dependent matrix metalloenzyme that enables collagen cross-linking thus creating a dense mesh of scar tissue [146]. LOXL2 is therefore an interesting target for the hepatic fibrosis treatment and numerous approaches to inhibit LOXL2 have been developed. Primarily, as it is copper-dependent enzyme, its activity can be impaired with the copper-binding ligands, such as D-penicillamine [145] and *β*-aminopropionitrile (BAPN) [147, 148]. Nevertheless, the most promising approach is the use of monoclonal anti-LOXL2 antibodies. They provide a specific allosteric inhibition of enzyme, by the binding with the scavenger receptor cysteine-rich (SRCR) domains, which are the catalytic center of the molecule [146, 147]. There were at least two types of antibodies reported: AB0023, murine monoclonal antibody against LOXL2, used in* in vivo* studies [146, 147] and Simtuzumab (SIM or GS-6624 and formerly AB0024), monoclonal antibody against human LOXL2. Simtuzumab is currently registered in 11 clinical trials (https://www.clinicaltrials.gov/), from which 6 are related to fibrotic liver diseases. The preliminary results of the pilot study in patients with liver fibrosis [149] reported that SIM was well tolerated at the applied doses (10 mg/kg) with no serious adverse effects and, due to its mechanism of action, provides a very promising antifibrotic therapy for patients with hepatic fibrosis. Additionally, another clinical trial has been launched recently evaluating simtuzumab in combination with GS-4997 (or selonsertib) in patients with nonalcoholic steatohepatitis (NASH) and fibrosis [150] (Table 1).

### 5.3. Targeted Conjugate

**Figure 2:**
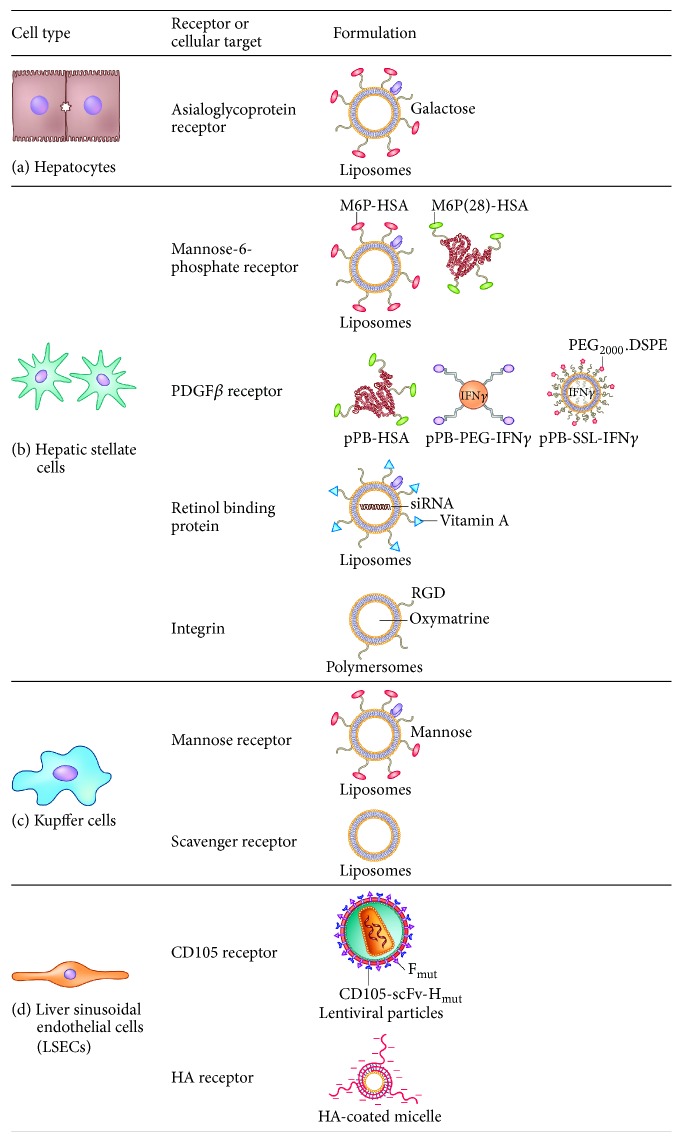
Receptors or cellular targets and different designed formulations for active targeting to the different cell types of liver. Nanoparticles or proteins are modified with specific surface ligands to be recognized by their receptors or cellular targets on a specific type of liver cells: (a) hepatocytes, (b) hepatic stellate cells (HSCs), (c) Kupffer cells (liver macrophages), and (d) liver sinusoidal endothelial cells.

Targeted conjugates are the most diverse and the newest from of the described approaches; however the concept is already more than a hundred years old, as is the idea of “magic bullet” by Paul Ehrlich [151]. The targeted conjugates are combining the features attributed to small molecule drugs and monoclonal antibodies. It can consist of the delivery vehicle, for example, protein carrier (HSA), liposome [152], polymeric nanoparticles, micelles, or nanoformulation, [153] containing the active component, such as small molecule drug [153], small interfering RNA (siRNA) [51], micro RNA (miRNA), cytokine [50], an active peptide, or a therapeutic protein. Targeting ligand is attached to guide the delivery carrier to the specific site in the body, based on the specific ligand-receptor interactions. This allows the preferential accumulation of the conjugate in specific target cells, tissues, or organs. Additional advantage of the targeted conjugates is that they provide the opportunity for theranostic approach (therapy and diagnostics). Application of the detectable moieties in the design, such as magnetic nanoparticles as the delivery vehicles or the fluorescent ligands on the surface of the conjugate, is to detect the accumulation of the particles in the target site. Moreover, magnetic nanoparticles can serve as the contrast agents in magnetic resonance imaging [154] and nanobubbles can provide the contrast enhancement in ultrasonography imaging [155]. There are many strategies and nanoformulations explored for the treatment of liver fibrosis in preclinical animal models. The strategies are developed to target different receptors on different liver cell types, that is, hepatocytes, hepatic stellate cells, Kupffer cells (liver macrophages), and liver sinusoidal endothelial cells [156]. These strategies are detailed in Table 2 and illustrated in Figure 2.

The only representative of targeted conjugate in targeted therapies against liver fibrosis and a very promising drug which is currently under investigation in phase 1b/2 clinical trial is vitamin A-coupled lipid nanoparticle (liposome) containing siRNA against collagen-specific chaperone heat shock protein 47 (HSP47) [3, 157, 158]. HSCs expresses retinol binding protein (RBP) receptor that regulates retinol (vitamin A) storage in HSCs and is an interesting target for HSC-specific drug delivery. HSC-targeted liposomes (ND-L02-s0201) carry siRNA against HSP47, which facilitates collagen secretion by ensuring triple-helix procollagen formation, and are implicated in translational regulation of procollagen synthesis [159, 160]. Downregulation of collagen production can result in the amelioration of fibrosis and reversion of cirrhosis [51]. Recently, Lawitz et al. [161] presented the preliminary results from the clinical trials performed on healthy subjects as well as on the patients with advanced liver fibrosis. ND-L02-s0201 was well tolerated in both groups of subjects without dose limiting toxicity neither in a single administration nor in multiple doses. Furthermore, in the liver fibrosis patients, 6 out of 8 patients showed at least 1-stage improvement in the liver fibrosis suggesting beneficial effects of targeted approach.

## 6. Conclusions

As presented in this review, the development of the targeted therapies against fibrotic diseases is in relatively advanced stage. Numerous drugs are being assessed in the phase 2 clinical trials while some of them also reached phases 3 and 4. Multiple studies are currently ongoing, and the already completed trials revealed high potential of emerging drugs in ameliorating hepatic fibrosis of various etiology. However, besides the already investigated mechanisms and drugs, there are still some target proteins and pathways that remain to be elucidated. Numerous promising molecular targets are currently under preclinical investigation and will be evaluated in the clinical trials. Nevertheless, taken all together, there is remarkable improvement in the development of targeted therapies against fibrotic diseases and, in noninvasive technologies, many drugs are already being tested but many exciting targets still remain to be explored and further investigated. It is giving hope for the patients that clinically approved efficacious treatment will emerge soon.

## Figures and Tables

**Table 1:** Summary of the registered clinical trials (Clinicaltrials.gov).

Drug type	Disease condition	Phase	Study type	Trial number
*Small molecule drugs*				
Farglitazar (GI262570), PPAR*γ* agonist	Liver fibrosis with chronic HCV infection	2	Safety/efficacy study	NCT00244751
Pioglitazone, PPAR*γ* agonist	NASH	2	Safety/efficacy study	NCT01068444
Pioglitazone, PPAR*γ* agonist + vitamin E	NAFLD with diabetes mellitus type 2 (T2DM)	4	Efficacy study	NCT01002547
Pioglitazone, PPAR*γ* agonist + vitamin E	Nondiabetic patients with NASH	3	Efficacy study	NCT00063622 (PIVENS)
Pioglitazone, PPAR*γ* agonist	Hepatic steatosis in HIV/HCV infections	4	Efficacy study	NCT00742326
Obeticholic acid, FXR agonist	NASH fibrosis	3	Efficacy study	NCT02548351
Obeticholic acid, FXR agonist	Primary biliary cirrhosis	3	Safety/efficacy study	NCT02308111; NCT01473524
Obeticholic acid, FXR agonist	NASH	2	Efficacy study	NCT01265498
Obeticholic acid, FXR agonist	Primary sclerosing cholangitis	2	Safety/efficacy study	NCT02177136
Obeticholic acid, FXR agonist + ursodeoxycholic acid (URSO)	Primary biliary cirrhosis	2	Safety/efficacy study	NCT00550862
Losartan, angiotensin II type 1 receptor antagonist	Liver fibrosis (F2-F3) with chronic HCV infection	4	Efficacy study	NCT00298714
Losartan, angiotensin II type 1 receptor antagonist	NASH	4	Efficacy study	NCT01051219
Irbesartan, angiotensin II type 1 receptor antagonist	Liver fibrosis with chronic HCV infection	3	Efficacy study	NCT00265642
Moexipril, angiotensin I converting enzyme	Primary biliary cirrhosis	2	Safety/efficacy study	NCT00588302
Candesartan, angiotensin II type 1 receptor antagonist	Alcoholic liver fibrosis	1 + 2	Safety/efficacy study	NCT00990639
Candesartan, angiotensin II type 1 receptor antagonist	Liver fibrosis with chronic HCV infection	2	Safety/efficacy study	NCT00930995
Glycyrrhizin, antioxidant	Chronic hepatitis C and F2/F3 liver fibrosis	3	Efficacy study	NCT00686881
Warfarin, anticoagulant	Liver fibrosis	2	Safety/efficacy study	NCT00180674
Galectin-3 inhibitor (GR-MD-02)	NASH with advanced fibrosis	2	Safety/efficacy study	NCT02421094
Galectin-3 inhibitor (GR-MD-02)	Portal hypertension in NASH with cirrhosis	2	Safety/efficacy study	NCT02462967
Pentoxifylline, TNF*α* suppressing phosphodiesterase inhibitor	Primary biliary cirrhosis	2	Safety/efficacy study	NCT01249092
Pentoxifylline, TNF*α* suppressing phosphodiesterase inhibitor + vitamin E	Liver fibrosis with chronic HCV infection	3	Efficacy study	NCT00119119
Pentoxifylline, TNF*α* suppressing phosphodiesterase inhibitor	NASH	2/3	Safety/efficacy study	NCT00267670
Pentoxifylline, TNF*α* suppressing phosphodiesterase inhibitor	NASH	2	Efficacy study	NCT00590161
S-adenosyl methionine (SAMe) versus pentoxifylline	NASH	2	Efficacy study	NCT02231333
Cenicriviroc, CCR2 and CCR5 antagonist	NASH	2	Safety/efficacy study	NCT02217475
Fuzheng Huayu, herbal medicine	Liver fibrosis with chronic HCV infection	2	Efficacy study	NCT00854087
Sorafenib, tyrosine kinase inhibitor	Liver cirrhosis with portal hypertension	2	Efficacy study	NCT01714609
Erlotinib, EGFR TK inhibitor	Liver cirrhosis with HCC resection	2	Safety/efficacy study	NCT02273362
Everolimus, mammalian target of rapamycin inhibitor	Liver fibrosis in posttransplant and recurrent HCV patients	2/3	Safety/efficacy study	NCT00582738, NCT01888432

*Monoclonal antibodies*				
Simtuzumab, humanized monoclonal antibody against lysyl oxidase-like-2	NASH with advanced liver fibrosis	2	Safety/efficacy study	NCT01672866
Simtuzumab, humanized monoclonal antibody against lysyl oxidase-like-2	Liver fibrosis with hepatitis C, HIV, HIV/HCV coinfection	2	Safety/efficacy study	NCT01707472
Simtuzumab, humanized monoclonal antibody against lysyl oxidase-like-2	Liver fibrosis with primary sclerosing cholangitis (PSC)	2	Safety/efficacy study	NCT01672853
Simtuzumab, humanized monoclonal antibody against lysyl oxidase-like-2 + Selonsertib (GS-4997)-apoptosis signal-regulating kinase 1 (ASK1) inhibitor	NASH and fibrosis stages F2-F3	2	Safety/efficacy study	NCT02466516
FG-3019, Human monoclonal antibody against connective tissue growth factor	Liver fibrosis with chronic hepatitis B infection	2	Safety/efficacy study	NCT01217632

*Targeted conjugate*				
Targeted liposome delivering siRNA against HSP47 (ND-L02-s0201)	Healthy subjects	1	Safety study	NCT01858935
Targeted liposome delivering siRNA against HSP47 (ND-L02-s0201)	Moderate to extensive hepatic fibrosis (F3-4)	1/2	Safety/efficacy study	NCT02227459

**Table 2:** Targeting strategies explored for the preclinical therapeutic treatment of liver fibrosis.

Cellular target	Targeting ligand	Carrier	Drug	References
*Hepatocytes*				
Asialoglycoprotein (ASGP) receptor	Galactose, galactosylated lipid (lactobionic acid)	Liposomes, solid Lipid nanoparticles	Quercetin, Cucurbitacin B, TLR4 siRNA	[123–125]

*Hepatic stellate cells*				
Mannose-6-phosphate receptor	Mannose-6-phosphate	HSA, liposomes	Doxorubicin, pentoxifylline, rosiglitazone, 15dPGJ2, Gliotoxin, Losartan, Y27632, rho-kinase inhibitor, ALK5 inhibitor LY-36947	[126–133]
Retinol binding protein (RBP)	Vitamin A	Liposomes, RcP nanoparticles	HSP47 siRNA, antisense oligonucleotides (ASO)	[51, 134]
Platelet-derived growth factor receptor	Cyclic peptide C^*∗*^SRNLIDC^*∗*^ and bicyclic peptide	HSA, peptide, liposomes	Interferon gamma (IFN*γ*) and mimetic IFN*γ*	[50, 135, 136]
Integrins	RGD peptide	Liposomes, polymersomes	Interferon alpha 1 beta (IFN-*α*-1b), hepatocyte growth factor, oxymatrine	[137–139]

*Kupffer cells (macrophages)*				
Mannose receptor	Mannose	Liposomes, nanoparticles	Dexamethasone, TNF*α*-siRNA	[140, 141]
Scavenger receptor	—	Liposomes	Dexamethasone	[142]

*Liver sinusoidal endothelial cells (LSECs)*				
Endoglin (CD105) receptor	Endoglin (CD105)	Lentiviral particles	Erythropoietin gene	[143]
Hyaluronic acid (HA) receptor	Hyaluronic acid	Micelles	—	[144]
